# Generation of human gastric assembloids from primary fetal organoids

**DOI:** 10.1007/s00383-023-05586-9

**Published:** 2023-11-24

**Authors:** Giada Benedetti, Brendan C. Jones, Francesca Sgualdino, Paolo De Coppi, Giovanni Giuseppe Giobbe

**Affiliations:** 1 Stem Cell and Regenerative Medicine Section, Great Ormond Street Institute of Child Health, University College London, London, UK; 2 Department of Specialist Neonatal and Paediatric Surgery, Great Ormond Street Hospital, London, UK

**Keywords:** Assembloid, Gastric organoid, Fetal stem cell, Stomach

## Abstract

**Purpose:**

Understanding human gastric epithelium homeostasis remains partial, motivating the exploration of innovative in vitro models. Recent literature showcases the potential of fetal stem cell-derived organoids in developmental and disease modelling and translational therapies. To scale the complexity of the model, we propose to generate assembloids, aiming to increase gastric maturation to provide new structural and functional insights.

**Methods:**

Human fetal gastric organoids (fGOs) were expanded in 3D Matrigel cultures. Confluent organoid cultures were released from the Matrigel dome and resuspended in a collagen I hydrogel. Subsequently, the organoid mixture was seeded in a ring shape within a 24-well plate and allowed to gelate. The structure was lifted in the medium and cultured in floating conditions, allowing for organoid self-assembling into a gastric assembloid. After 10 days of maturation, the assembloids were characterized by immunostaining and RT-PCR, comparing different fetal developmental stages.

**Results:**

Successful generation of human fetal gastric assembloids (fGAs) was achieved using spontaneous self-aggregation within the collagen I hydrogel. Immunostaining analysis of early and late fGAs showed the establishment of apico-basal cell polarity, secretion of gastric mucins, and the presence of chromogranin A in both samples. Transcriptional markers analysis revealed distinct disparities in markers associated with mature cell types between late and early fetal stages.

**Conclusions:**

fGOs can reliably be generated from human fetal samples. This pioneering assembloid approach paves the way for advancing our comprehension of human gastric epithelium homeostasis and its perturbation, offering a better in vitro platform for the study of gastric epithelial development and therapeutic translation.

## Introduction

Gastrointestinal organ failure might largely benefit of regenerative medicine applications, as either congenital or postnatally acquired pathologies relying on transplantation for treatment, which is associated to a variety of complications, particularly in children [1]. Regenerative medicine stands as a cutting-edge research field, holding the promise of addressing unmet medical needs, integrating diverse disciplines, including cell and molecular biology, clinical research, biomedical and biomaterial engineering [2, 3].

In this context, the role of organoids emerges as a pivotal component in regenerative medicine. They represent an invaluable in vitro model, mirroring structures and functions proper of organs. Still, the understanding of human gastric epithelium homeostasis remains partial, encouraging the exploration of innovative research models. Indeed, while many of the studies concerning stomach development have been carried out in rodents [4–8], this knowledge has led to the successful generation of human gastric organoids through direct differentiation from induced pluripotent stem cells [9, 10]. The need of shorter protocols and the possibility of tailoring treatment at patient level, has brought forward the urge of primary derived organoids [11–14]. These first studies were performed by isolating stem cells from adult biopsies. Current literature showcases the potential of fetal stem cell-derived organoids in disease modelling and translational therapy platforms [15].

Recently, bulk and single-cell RNA sequencing approaches have been applied to human fetal endodermal organs [16]. Thanks to this approach, the authors gained insights on cell populations that compose the fetal stomach throughout development (form PWC 6 to PWC 24). Further insights were reported by Giobbe et al., who analysed gastric fetal tissues, and for the first time also the organoids derived from gastric fetal tissues (fGOs), at early (8 and 11 PWC) and late (18 and 20 PWC) stages of development, showing fGOs to have coherent patterns of expression compared to the tissue of origin [15].

The use of primary fGOs offers many unprecedented benefits to study human development and congenital diseases. This approach brings into focus the potential to unravel insights into early organ development and disease processes while addressing issues of expandability and applicability to post-natal stomach research.

**Fig. 1:**
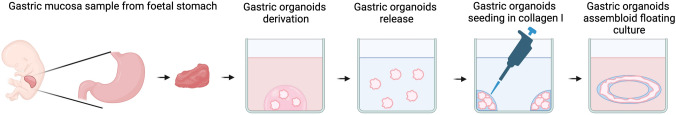
Schematic representation of the workflow for isolating human fetal gastric stem cells and derivation of organoid lines for generating gastric fetal assembloids. Created with BioRender.com

However, the 3D configuration and differentiation capacity of fGOs is still limited. Therefore, we here propose an increase in complexity by scaling up the size of the in vitro model. In particular, we propose to generate an assembloid by allowing the self-aggregation of primary-derived fGOs (Fig. 1). This protocol was performed on organoids derived from different fetal stages, to allow us to spot any relevant difference according to the developmental stage. With this adaptation of the epithelial organoid system [17], we aim to provide a large-scale gastric platform to allow new structural and functional insights, to study either gastric epithelial homeostasis and disease during development or for therapeutic translation.

## Materials and methods

### Ethic statement

Human fetal stomachs were dissected from tissue obtained immediately after termination of pregnancy from 8 to 21 post-conception week (PCW), in compliance with the bioethics legislation in the UK. Fetal samples were sourced following patient informed consent via the Joint MRC/Wellcome Trust Human Developmental Biology Resource with Research Tissue Bank ethical approval (08/H0712/34+5).

### Organoid culture

Organoid lines were derived from human fetal stem cells as previously described [15]. Organoids were passaged in culture weekly [15]. Dissociation to single cells allowed for rapid expansion in organoid number [18]. Organoids were collected by scraping the plate and washed in ADMEM/F12 (Thermo Fisher, 12,634) plus 1% penicillin–streptomycin (Thermo Fisher, 15140122), 10 mM HEPES (Thermo Fisher, 15630080), and 2 mM Glutamax (Thermo Fisher, 35050061) (called “ADEM/F12+++”). The cell pellet was then resuspended in 1 mL of TrypLE Express (Thermo Fisher, 12605010) and incubated for 5 min at 37 °C. Organoids were then further pipetted, and 10 mL of was added to inactivate the TrypLE Express. Cells were centrifuged and the pellet was dried. Near-dry pellets were resuspended in the appropriate volume of Matrigel^®^ (Corning, 354230) plated in 30 μL droplets, plate was inverted and the drops allowed to gelate for 30 min in incubator. fGO medium was added and changed every 3 days. Human gastric organoid medium includes ADMEM/F12+++ as above), 1X B-27 supplement without vitamin A (Thermo Fisher, 12587010), 1.25 mM N-acetylcysteine (Sigma Aldrich, A9165), 100 ng/mL Wnt-3A (Peprotech, 315–20), 500 ng/mL R-spondin 1 (Peprotech 120–38), 100 ng/mL Noggin (R&D Systems, 6057-NG), 50 ng/mL epidermal growth factor (EGF) (Thermo Fisher, PMG8043), 10 nM gastrin (Sigma Aldrich, G9020), 3 μM glycogen synthase kinase 3 (GSK-3) inhibitor (CHIR99021) (Tocris, 4423), 5 μM transforming growth factor beta (TGFβ) inhibitor (A83-01) (Sigma Aldrich, SML0788), and 200 ng/mL fibroblast growth factor 10 (FGF10) (Peprotech, 100–26) [19]. 10 μM of Rho kinase inhibitor Y-27632 was added to the medium after plating, but not during subsequent medium changes (every 3 days).

### Assembloid formation and culture

The following methodology was based on a modified protocol by Sachs et al. [17]. Pure rat tail collagen I was used to prepare a 0.75 mg/mL collagen I (First Link, 60-35-810) hydrogel of physiological pH and salinity (1X Advanced DMEM/F12, Thermo Fisher, 12500062; 10 mM HEPES, Thermo Fisher, 15630080; 0.75 mg/mL Collagen I, First Link, 60–35-810; in MilliQ water ZIQ7003T0; pH 7.5). The hydrogel was prepared fresh for each experiment and kept on ice until combined with organoids. On day 7 after seeding as single cells, whole organoids were release from Matrigel using Cell Recovery Solution™ (Corning, 354253) for 45 min on ice, and collected in a 1% BSA coated 1.5 mL tube. Organoids were pelleted at 100 g for 5 min. An ice-cold PBS wash was performed to remove any residual Matrigel^®^. Organoids were resuspended in the appropriate volume of Collagen I solution prepared as mentioned above (200 μL per well), and seeded in an ultra-low attachment 24-well tissue culture plate (Corning, 3473) in a ring shape. The plate was incubated at 37 °C for 30 min. The assembloid was then detached from the bottom of the well by forceful addition of 500 μL of complete medium, followed by a further 500 μL of complete medium (total 1 mL per well). Medium was changed every 1/3 days, depending on cell replication, until conclusion of the experiment at day 10. A total of six droplets were used per 200 μL of collagen I.

## Organoids whole-mount immunofluorescence staining

fGOs at 7 days from last passage were removed from Matrigel^®^ by incubation in Cell Recovery Solution for 45 min at 4 °C. This treatment released the organoids from the Matrigel^®^ without damaging the structure of the organoids, allowing whole mount immunofluorescence staining. Organoids were collected, washed in PBS, and transferred to 1% BSA pre-coated 1.5 mL Eppendorf tubes, and resuspended in 4% PFA (PFA; Sigma-Aldrich, 100496) for 20 min in rotation. PFA was removed and quenched with 0.1 M NH_4_Cl (Sigma-Aldrich, 254134) for 1 h in rotation to decrease aldehyde-related autofluorescence. Quenched organoids were stored in PBS with 1% penicillin–streptomycin until staining.

fGAs were first fixed in 4% PFA for 30 min, washed with PBS, and residual PFA quenched with 0.1 M ammonium chloride (NH_4_Cl; Sigma-Aldrich, A9434) for 60 min to quench unreacted aldehyde groups and reduce autofluorescence. Samples were then dehydrated overnight in 30% sucrose (Sigma-Aldrich, S9378), before embedding in Polyfreeze Tissue Freezing Medium (Polysciences, 25113) over dry ice, and then sectioned at 7 μm on a Bright OTF cryostat. Sections were stored at − 20°C until staining. After defrosting, sections were re-fixed to the slide with 5 min of 4% PFA, quenched with 15 min of 0.1 M NH_4_Cl, and washed with PBS.

fGOs and fGAs were blocked and permeabilised with 0.5% Triton X-100 (Sigma-Aldrich, 100496) with 1% BSA in PBS for 2 h (fGOs) or 1h (fGA sections) at room temperature.

Primary antibodies were diluted in blocking buffer and incubated with sections or organoids for 24 h at 4 °C in rotation. Samples were then extensively washed in 0.5% Triton X-100 in PBS. Secondary antibodies were diluted and applied to samples as for primary antibodies and incubated overnight at 4 °C. fGOs in suspension were transferred to a glass-bottomed Petri dish immediately prior to imaging. fGAs’ sections were mounted in non-DAPI mounting medium (Biorad, BUF058A).

Primary antibodies: Chromogranin A (Abcam, ab15160), Mucin 5AC (Invitrogen, ma5-12178), Mucin 6 (Abcam, ab216017), Ki67 (Abcam, ab15580), Integrin beta-4 (INTB4) (Abcam, ab110167) all at 1 in 100 dilution. Ezrin (EZR) (thermos Fisher, PA5-29358) at dilution 1:200.

Secondary antibodies were AlexaFluor^®^ anti-mouse 488 (Thermo Fisher, A11001), AlexaFluor^®^ anti-rabbit 568 (Thermo Fisher, A11011), anti-Rat 594 (Thermo Fisher, A11007) all at 1 in 200 dilution. AlexaFluor^®^ Phalloidin 647 (Thermo Fisher, A22287) was used at 1:100 dilution. Hoechst 33342 (Thermo Fisher, H1399) at 10 μg/mL.

### Image acquisition

Whole mount stainings were acquired on the Zeiss LSM 710 confocal microscope. Overview images were acquired using the Zeiss Plan-Apochromat 10x, NA 0.45 mm, dry (air) objective (working distance: 2 mm) with tile scanning. Brightfield images of organoids in culture were acquired using on a Zeiss Axio Observer A1 inverted widefield microscope with a Colibri 5 LED light source and Axiocam MRm camera system.

### RNA extraction and reverse transcription

fGAs were removed from the culture well and excess collagen I hydrogel was trimmed from the edges with a scalpel under a dissecting microscope. fGAs were then minced with a scalpel in a petri dish on ice prior to transfer to lysis buffer. RNA extraction was done using Qiagen RNeasy Micro (74004) kit. RNA was extracted following the manufacturers recommendations. RNA quantified by spectrophotometry using the NanoDrop 1000 (Thermo Fisher Scientific) and immediately reverse transcribed to cDNA or stored at − 80 °C. Reverse transcription was performed with the High-Capacity cDNA Reverse Transcription Kit (Applied Biosystems, 4368814) according to the manufacturer’s instructions.

### Real-time PCR (RT-PCR) analysis

Quantitative real-time PCR was performed using the Applied Biosystems™ TaqMan^®^ gene expression assays (all from Thermo Fisher Scientific) according to manufacturer’s instructions in 96-well PCR plates. The TaqMan^®^ FAM-labelled probes were used according to the manufacturer’s instructions. The following probes (all from Thermo Fisher) were used: GAPDH (glyceraldehyde 3-phosphate dehydrogenase), LGR5 (leucine-rich repeat-containing G-protein coupled receptor 5), AXIN2 (axin-like protein), MUC5AC (mucin 5AC), MUC6 (mucin 6), PGA5 (pepsinogen A5), SST (somatostatin), GAST (gastrin), CHGA (chromogranin A). Glyceraldehyde 3-phosphate dehydrogenase (GAPDH) was used as endogenous control in each well with the VIC reporter. The plate was loaded on a StepOnePlus Real Time PCR System running StepOne Version 2.3 software (Applied Biosystems, 4376600) for reactions. Data were downloaded to Microsoft Excel for Mac (Microsoft, Version 16.55) for analysis. RT-PCR data were analysed using the ΔΔ*Ct* method. Relative fold change was graphically represented as individual data points and mean ± standard error of the mean (SEM), using GraphPad Prism for Mac (Version 10).

## Results

### Fetal gastric organoids can self-organize into a complex assembloid.

**Fig. 2:**
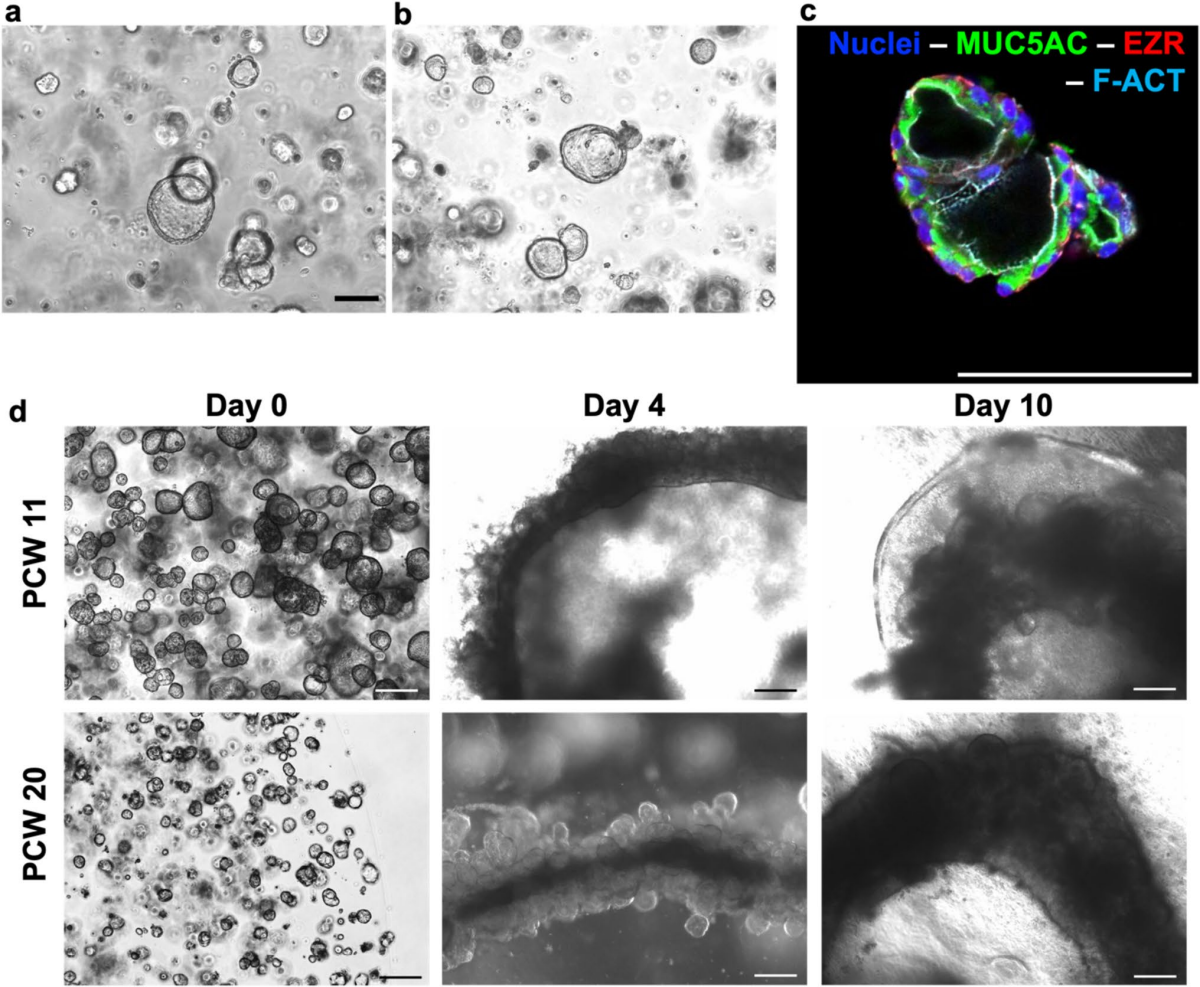
**a**, **b** 6-days grown gastric fetal organoid 3D culture at passage 2 (PWC 11 **a**, PCW 20 **b**). Scale bar 100 μm. **c** Immunofluorecense image of gastric fetal organoids. Mucin 5AC (MUC5AC) in green, Ezrin (EZR) in red, F-actin (F-ACT) in cyan, and nuclei (Hoechst) in blue. Scale bar 100 μm. **d** Assembloid formation: brightfield picture at day 0 (whole organoids after seeding), day 4, and day 10 self-assembly, for both organoids lines (PCW 11 and PCW 20). Scale bar 200 μm

As a first step for the assembloid generation protocol, we cultured 3D human fGO cultures derived from isolated stomach stem cells, as previously described by our group [15]. For this purpose, we used two different fetal stages: PCW 11 (defined early fetal stage) (Fig. 2a) and PCW 20 (defined late fetal stage). Immunofluorescence analyses was conducted on the fGO lines and showed the presence of polarized epithelial cells, with F-actin underlying the apical (inner) pole. The marker ezrin delimits the epithelial cells and play a major role in organizing cortical domains by assembling membrane protein complexes with actin. Stomach marker mucin-5AC was shown in the luminal side of the organoids displaying the gastric specificity of the cells (Fig. 2c).

Fully formed fGOs were used as the starting point of fGA generation. In particular, single-cell dissociated fGOs were seeded single cell in Matrigel drops, and allowed to grow for 7 days. fGOs were released from the Matrigel dome, and appropriately resuspended in a collage I hydrogel. Around 12,000–15,000 organoids/mL density was used to seed a 200 μL pre-gel solution as a circumferential ring on the bottom of a 24-well plate. The hydrogel was allowed to gelate, and consequently lifted by medium flushing. This setting allowed the organoids to remodel and contract the collagen, enabling self-aggregation and fusion into a complex fGA structure in a 10-day time window. Separated fGOs were visible in the collagen hydrogel at day 0 (right after seeding). By day 4, a premature fused structure was visible, with single fGOs still visible amidst the tubular shaped macrostructure. By day 10, a complete fGA ring reliably formed a single tubular structure (Fig. 2d).

### Fetal gastric assembloid promote epithelial differentiation

**Fig. 3:**
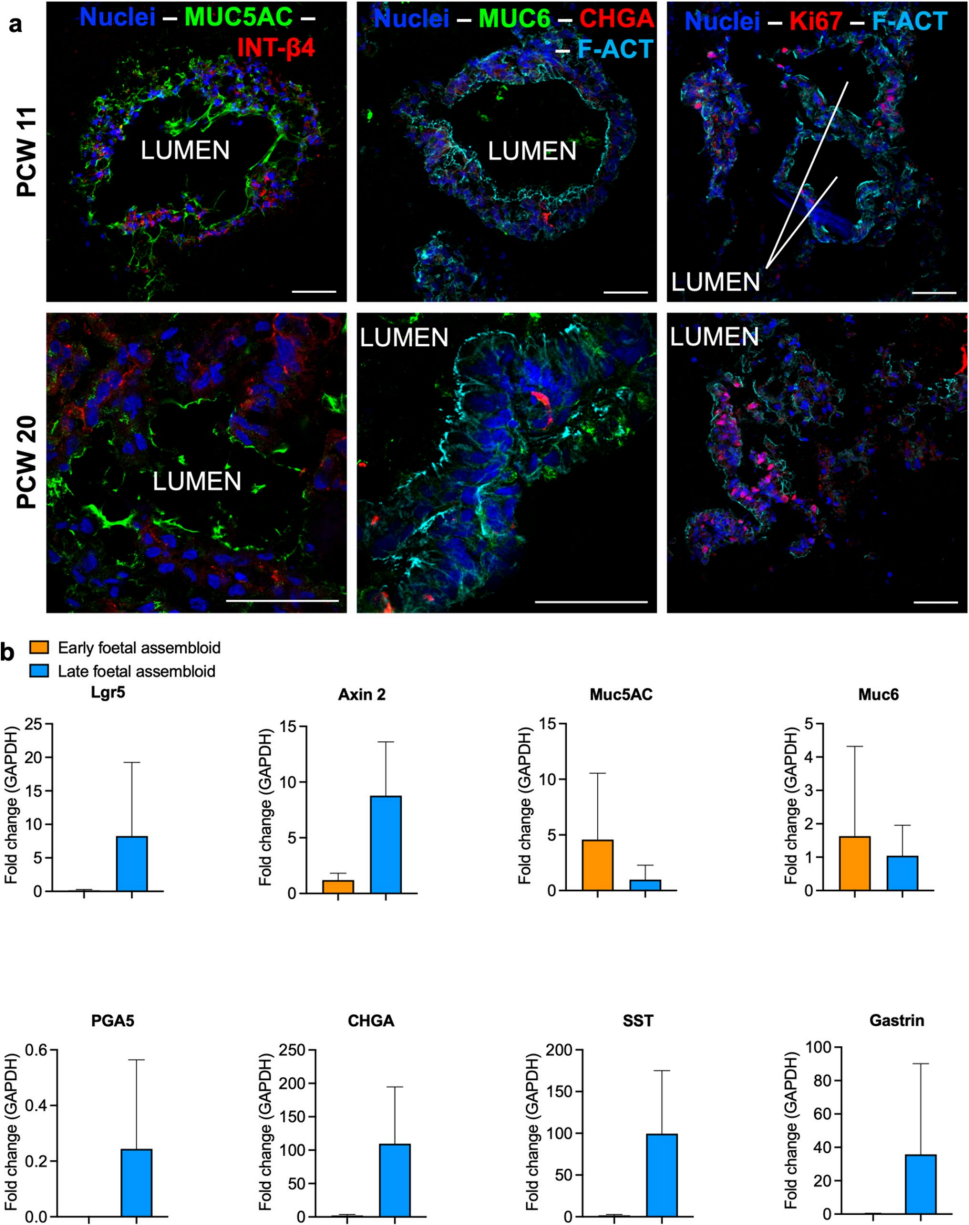
**a** Immunofluorescence images of sectioned gastric assembloids derived from early fetal or late fetal organoids. The label LUMEN identifies where the inner portion of the assembloid. Mucin 5AC (MUC5AC) in green, Integrin β4 (Int-β4) in red. Mucin6 (MUC6) in green, Chromogranin A (CHGA) in red, F-actin (F-ACT) in cyan, Ki67 in red, nuclei (Hoechst) in blue. Scale bar 50 μm. **b** RT-PCR characterization of stem cell and gastric mature marker expression in fetal assembloids. Relative fold change to GAPDH, normalised to the Matrigel-cultured organoids from the same developmental stage (*n* = 3 for each developmental stage)

Initial characterisation of the fGA was performed by immunofluorescence analysis on sectioned assembloids. We could observe the presence of a continuous cell layer around the luminal side. Sections of the fGAs demonstrated the presence of gastric mucin 6 and mucin 5AC, as well as CHGA^+^ enteroendocrine cells, coherently with the fact that these are the first mature cell types to emerge at tissue level [15]. Apical-in cell polarity appeared to be preserved in the sections examined, and Ki67 staining showed the persistence of a proliferative cell compartment in the fGAs (Fig. 3a). No quantifiable immunofluorescence differences were observed between early and late stage fGAs. Fetal gastric assembloids were then characterised by RT-PCR for stem and mature gastric cell markers. fGA gene expression profiles were compared between early and late fetal stages by normalizing these to the fGOs of the corresponding developmental stage. While high biological variability was observed, there were some interesting findings. Early fGAs displayed a trend towards downregulation of stem markers, and upregulation of mucins (*MUC5AC* and *MUC6*). In contrast, late fGAs tended towards upregulation of stem (*LGR5, AXIN2*), and enteroendocrine cell markers (*CHGA, SST, GAST*), compared to the organoids of the corresponding developmental stages (Fig. 3b). *PGA5* appeared to be expressed only in late fGAs, consistent with its absence during early developmental stages in the tissue as well [15].

## Discussion and conclusion

As previously shown by our research group, organoids obtained from surgical specimens are a tremendous tool for recapitulating organized gastric epithelia, showing apico-basal polarity and the presence of certain mature cell types [20]. With the introduction of our assembloid model, we further increased the complexity of the system by scaling up in dimension and allowing the spontaneous aggregation of simpler organoids.

This study marks a significant advancement in the field of regenerative medicine and organoid research, with the successful generation of functional fGAs starting from fGOs from early and late developmental stages. Over a 10-day maturation period, the assembloids exhibited consistent self-organization capacity in all experiments performed, underscoring their protocol reliability.

Regarding fGA characterisation, the trends in differential expression of some key gastric genes, such as mucins, enteroendocrine markers (*CHGA*, *SST*, *GAST), PGA5,* suggests the potential of this model to investigate changes in the gastric epithelium maturation according to developmental stages. By increasing the complexity of the organoid in vitro model, we have provided assembloid tool model that is more reliable and can facilitate mechanical studies on the early formation of the stomach sac [21].

Furthermore, the increase in dimension and complexity of the assembloid compared to single organoids provides easier access to the lumen, simplifying the testing of specific treatments or facilitating host–pathogen interaction studies. This eliminates the need for complex microinjection systems currently used with organoids [12].

This in vitro model opens exciting possibilities for gaining insight into related developmental pathologies, such as microgastria [21], pyloric atresia [21, 22], or congenital syndromes with a broad spectrum of symptoms, which also affects the stomach [23, 24]. Moreover, it could be of particular relevance for studying in vitro other more common paediatric and adult diseases such as gastroesophageal reflux disease and the dysregulation which occurs with acid production. The gastric assembloid holds the potential of modelling congenital mucosal diseases, as well as hereditary or acquired cancer types, and investigating the molecular mechanism underlying these disorders. Additionally, they have the benefit of being easily obtainable in an autologous setting and, therefore, the derived assembloids could be applied as a platform for personalized medicine treatments to be tailored to the patient’s genetic background.

Looking ahead, there is tremendous potential to enhance the complexity of this system even further. Incorporating mesenchymal cell types, vascularization and enteric nervous system components could help better mimic physiological condition, enabling the modelling, study, and treatment of a broader spectrum of diseases.

In conclusion, our study has successfully demonstrated for the first time the generation of gastric assembloids using fetal organoids. Our protocol allows the organoids to self-organise into macrostructures with continuous lumen while retaining the expression patterns and normal polarity of the tissue of origin. This innovative approach introduces gastric assembloids as a novel and versatile system for studying development, disease mechanisms, and personalized medicine treatments.

## Data Availability

The authors declare that all data supporting the findings of this study are available within the article, main figures and figure legends, or from the authors upon reasonable request.
